# Amelioration of Androgenetic Alopecia by Algal Oligosaccharides Prepared by Deep-Sea Bacterium Biodegradation

**DOI:** 10.3389/fmicb.2020.567060

**Published:** 2020-09-29

**Authors:** Min Jin, Yu-Lei Chen, Xiongfei He, Yanping Hou, Zhuhua Chan, Runying Zeng

**Affiliations:** ^1^Third Institute of Oceanography, Ministry of Natural Resources, Xiamen, China; ^2^Southern Marine Science and Engineering Guangdong Laboratory, Zhuhai, China; ^3^College of Food and Biological Engineering, Jimei University, Xiamen, China; ^4^Aquabrain Biotech (Xiamen) Co., Ltd., Xiamen, China

**Keywords:** androgenetic alopecia, algal oligosaccharides, dihydrotestosterone, deep-sea bacterium, anagen

## Abstract

Androgenetic alopecia (AGA) is a dihydrotestosterone (DHT)-mediated hair loss disorder characterized by shortened anagen hair cycle. Oligosaccharides derived from seaweeds possess diverse biological functions. However, little is known about their effects on AGA. In this study, algal oligosaccharide (AOS) was characterized for its mitigation effects on key features involved in AGA pathogenesis, such as DHT- mediated cellular signaling and shortened anagen hair cycle. AOS with varying degrees of polymerization (DP), namely, AOS (DP2), AOS (DP4–6), and AOS (DP8–12), were prepared by agar biodegradation with *Flammeovirga pacifica* WPAGA1, an agarolytic bacterium isolated from deep-sea sediments. *In vitro* results showed that AOS with varying DPs significantly ameliorated the DHT-induced alterations of regulatory factors in human hair follicle dermal papilla cells in a dose- and DP-dependent manner, as revealed by the normalization of several hair-growth-stimulating or inhibitory factors. *In vivo* studies showed that AOS (DP2) extended the anagen phase and thereby delayed catagen progression in mice. Furthermore, AOS (DP2) stimulated dorsal hair growth in mice by increasing hair length, density, and thickness. Therefore, our findings indicated that AOS antagonized key factors involved in AGA pathogenesis, suggesting the potential application of AOS in the prevention and the treatment of AGA.

## Introduction

Androgenetic alopecia (AGA), commonly known as male pattern baldness, is an androgen-dependent hair loss disorder characterized by the progressive transformation of scalp terminal hairs into vellus hairs (Motofei et al., 2018). AGA is the most common type of progressive hair loss disorder in men and women (Lolli et al., 2017). AGA generally occurs after puberty, and its prevalence and incidence depend on age and race. Approximately 41.4% of Chinese men aged more than 70 years are affected by AGA, and the overall prevalence of AGA in Chinese men is 21.3% (Wang et al., 2010). The prevalence of AGA is significantly higher in Caucasians than in Asians; more than 30% of white men will have AGA at age 30, and 80% will have the disorder at age 70 (Severi et al., 2003; Otberg et al., 2007). Although AGA is a benign condition, it can lead to negative psychological effects, which are associated with depression, anxiety, worries about aging, and low self-esteem, in men and women (Severi et al., 2003).

The known important causative factors involved in the pathogenesis of AGA are androgen, genetic factors, and age, although the complete molecular mechanisms have not been fully understood (Lolli et al., 2017). Androgen plays the most important role in the etiology of AGA. The main circulating androgen in men is testosterone. However, AGA appears more related to the most active androgenic compound, namely, dihydrotestosterone (DHT), which is converted from testosterone by 5α-reductase (Motofei et al., 2018). The affinity of androgen receptor (AR) for DHT is five times higher than that for testosterone. In hair follicles, AR is located in mesenchymal-derived dermal papilla cells, which are the action sites of DHT on scalp hair growth (Inui and Itami, 2011). Dermal papilla cells play fundamental roles in the induction and the maintenance of follicle epithelial cell growth and mediate the stimulating signals of DHT by secreting numerous growth factors and/or extracellular matrix factors on follicle cells (Botchkarev and Kishimoto, 2003; Blanpain et al., 2004; Roh et al., 2004). The released compounds have a paracrine effect on hair follicle epithelial cells as well as an autocrine effect on the dermal papilla itself (Lolli et al., 2017). Such factors include hair-growth-stimulating factors, such as vascular endothelial growth factor (VEGF) and insulin-like growth factor 1, and inhibitory factors, such as transforming growth factor beta 2 (TGF-β2) and Dickkopf 1 (DKK-1) (Blanpain et al., 2004; Lolli et al., 2017). DHT-induced alteration in regulatory paracrine factors will cause a progressive miniaturization of hair follicles, thereby shortening the anagen phase and eventually leading to a bald appearance (Pierard-Franchimont and Pierard, 2001; Inui et al., 2002; Lolli et al., 2017). Therefore, DHT-induced cellular signaling in dermal papilla cells and the subsequent shortening of anagen hair cycle serve as promising targets for AGA treatment.

Only two medications have been approved by the Food and Drug Administration to treat AGA, namely, minoxidil and finasteride. Although minoxidil has been used for more than 30 years to treat AGA, its precise mechanism of action on hair growth remains elusive (Messenger and Rundegren, 2004). Several primary mechanisms of minoxidil action are proposed: (a) minoxidil suppresses AR-mediated functions by decreasing AR transcriptional activity and reducing the expression of AR targets at the protein level (Hsu et al., 2014), (b) minoxidil exerts anagen prolongation effects by activating β-catenin pathway in dermal papilla cells (Kwack et al., 2011), (c) minoxidil stimulates hair follicle angiogenesis by inducing the expression of VEGF in dermal papilla cells (Lachgar et al., 1998), (d) minoxidil shows a cytoprotective activity by activating prostaglandin synthase-1 (Michelet et al., 1997), and (e) minoxidil increases blood flow by dilating hair follicle arteries (Messenger and Rundegren, 2004). Finasteride is a DHT synthesis inhibitor, which suppresses the conversion of testosterone to DHT by binding to 5α-reductase (Varothai and Bergfeld, 2014; Kelly et al., 2016; Adil and Godwin, 2017). Although minoxidil and finasteride have proven effective in ameliorating AGA with long-term daily use, their use is costly and leads to variable adverse effects that may even persist after treatment cessation. The most frequent adverse reactions related to minoxidil include scalp irritation, dryness, and allergic contact dermatitis (Tsuboi et al., 2012). The adverse effects of finasteride include mental/psychological impairment (lack of mental concentration, depression, suicidal ideations) and sexual disorders (decreased libido, erectile dysfunction, ejaculation disorder) (Motofei et al., 2016; Rowland et al., 2016). In this respect, alternative therapeutic or nutritional solutions for the treatment of AGA with less side effects should be identified. In the last two decades, algal oligosaccharides (AOS), especially oligosaccharides derived from agar (the major cell wall component of red algae), have been reported to possess diverse physiological and biological functions, such as anti-oxidation, liver injury protection, antitumor, immune modulation, and whitening and skin-moisturizing effects; hence, AOS have been an attractive compound in cosmetic, food, nutritional, and pharmaceutical industries (Enoki et al., 2010; Kim et al., 2010; Gao et al., 2017). In the present study, AOS with varying degrees of polymerization (DP) were prepared by agar biodegradation with *Flammeovirga pacifica* WPAGA1, a deep-sea agarolytic bacterium (Xu et al., 2012; Gao et al., 2017). Then, the mitigation effects of AOS on key features involved in AGA pathogenesis were characterized.

## Materials and Methods

### Preparation of AOS With Varying DPs

AOS with varying DPs were prepared by agar biodegradation with a combination of three agarases, i.e., Aga2133, Aga4383 (Hou et al., 2015), and Aga2660. These enzymes were isolated from *F. pacifica* WPAGA1, a deep-sea bacterium that efficiently degrades agar (Xu et al., 2012; Gao et al., 2017). The three agarases show distinct endolytic activities on agar degradation, yielding various intermediate and end products, which are useful to produce AOS with varying DPs. Agar was degraded by recombinant Aga2133 to produce AOS (DP 8–12), an intermediate product at the early stage of degradation. Recombinant Aga4383 was used to degrade agar to yield AOS (DP4–6) as end products. The purified AOS (DP4–6) were then further hydrolyzed by recombinant Aga2260 to generate AOS (DP2) as end products. During each degradation process, samples were collected at different reaction time points and were heated at 100°C for 10 min to terminate the reaction. After centrifugation at 15,000 g and 4°C for 10 min, the supernatant was examined for AOS content through thin-layer chromatography (TLC) by using the method of Hou et al. (2015). After verification, the collected supernatant containing the desired AOS was lyophilized as AOS samples (DP2, DP4–6, and DP8–12) for further analysis.

### Cell Culture and Treatment

Human hair follicle dermal papilla cells (hHFDPC) were obtained from Guangdong Biocell Biotechnology Co., Ltd. (Guangdong, China) and cultured in mesenchymal stem cell medium (ScienCell, United States) in a humidified atmosphere with 5% CO_2_ at 37°C. All cell subcultures were performed at 70–80% cell confluence, digested with 0.25% trypsin, and inoculated into six-well plates or 96-well plates according to the experiment design.

hHFDPCs were harvested and inoculated into six-well plates at a density of 3 × 10^5^ cells per well or into 96-well plates at a density of 10,000 cells per well and then cultured at 37°C for 24 h. The culture medium was added with 800 nM DHT to induce AGA-associated alterations in regulatory paracrine factors in hHFDPCs. Fresh culture medium alone was added as blank control. To assess the protective effects of AOS, an AOS sample of a given working concentration was added to the DHT-supplemented culture. The DHT-supplemented culture was added with 500 μM minoxidil as a positive treatment control. The cells were further cultured for 24 h and subjected to MTT assay, cytokine production measurement, or gene expression determination.

### MTT Assay

hHFDPC cells were harvested, inoculated into 96-well plates at a density of 10,000 cells per well, and cultured at 37°C for 24 h. The growth medium was discarded and replaced with fresh medium containing various concentrations of the samples. In each plate, wells with culture medium but without cells were used as blank controls, and wells containing cells with medium and solvent only were used as solvent control. After 24 h of exposure to the samples, the cells were washed twice with phosphate-buffered saline gently, incubated in serum-free medium containing 0.5 mg/ml MTT at 37°C for 4 h, and extracted with 150 μl of dimethyl sulfoxide per well. The colored solution was detected for optical density (OD) at a wavelength of 490 nm by using Multiskan MS Analyzer (BioTek Epoch, United States). Cell viability was calculated by the following formula: viability rate (%) = (OD value of sample – OD value of blank control)/(OD value of solvent control – OD value of blank control) × 100.

### Measurement of Secreted VEGF by ELISA

After 24 h of incubation, the culture supernatant was collected and centrifuged at 12,000 g and 4°C to remove cell debris. VEGF concentration in the culture supernatant from sample-treated hHFDPCs was detected using VEGF ELISA kits (Abcam, United States) following the manufacturer’s protocol. OD at 450 nm was recorded using Multiskan MS Analyzer (BioTek Epoch).

### Determination of the Expression of Hair-Growth-Related Genes by qPCR

Total RNA of hHFDPCs in each group was extracted by using RNAiso Plus Kit (TaKaRa, China). According to the manufacturer’s instructions, total RNA was converted into first-strand cDNA by using a PrimeScript^TM^ RT reagent kit (TaKaRa). Quantitative real-time PCR was conducted to determine the transcript levels of β-catenin, TGF-β2, DKK1, and AR by using SYBR Green Real time PCR Master Mix (TaKaRa). GAPDH was used as the reference gene. The gene-specific primers used in this assay are listed in Table 1. The reaction was performed in the CFX96 Detection System (Bio-Rad, Hercules, CA, United States). The result was calculated by the 2^–ΔΔ*Ct*^ method.

**TABLE 1:** Primers used in the qPCR assay.

Gene name	Forward primer (5′–3′)	Reverse primer (5′–3′)
β-catenin	TTAGCTGGTGGGCTGCAGAA	GGGTCCACCACTAGCCAGTATGA
TGFβ2	TTACACTGTCCCTGCTGCACTT	GGTATATGTGGAGGTGCCATCAA
DKK1	ATGCGTCACGCTATGTGCTG	TGGAATACCCATCCAAGGTGCTA
AR	CTCTCACATGTGGAAGCTGCAAG	TTTCCGAAGACGACAAGATGGAC
GAPDH	GCACCGTCAAGGCTGAGAAC	TGGTGAAGACGCCAGTGGA

### *In vitro* Transdermal Permeability Experiments of AOS

The abdomen skin of Sprague–Dawley rats was fixed in a Franz diffusion pool. The donor solution was 500 μl of different AOS formulas (seven groups, Table 2). The receiving tank was added with 6 ml of physiological saline, and the water bath was equilibrated to 37°C at a fixed speed of 300 rpm. Subsequently, 1 ml of each sample was withdrawn and immediately replaced with an isothermal and equal volume of receiving solution at different time points (0.5, 1, 3, 4, and 5 h). The amount of AOS (DP2) in the sample was determined by the dinitrosalicylic acid method. The cumulative permeation per area of the skin (*Q*_*n*_, μg/cm^2^) was calculated according to the following equation, with the *in vitro* transdermal release curve plotted with time (hour) as the abscissa and *Q*_*n*_ (μg/cm^2^) as the ordinate. The experiment was repeated three times.

$$Q_{n}=V_{n}C_{n}+\Sigma i=1n-1C_{i}V_{i}A$$

**TABLE 2:** Experimental design of algal oligosaccharide (AOS) transdermal permeation-enhancing formula.

Group No.	Penetration-enhancing formula
1	1% AOS (DP2)
2	1% AOS (DP2) + 1% glycerol
3	1% AOS (DP2) + 2% glycerol
4	1% AOS (DP2) + 1% PEG400
5	1% AOS (DP2) + 2% PEG400
6	1% AOS (DP2) + 1% glycerol + 1% PEG400
7	1% AOS (DP2) + 2% glycerol + 1% PEG400

where *C*_*n*_ is the drug concentration in the endothelial compartment at different times (mg/ml), *C*_*i*_ is the drug concentration in the samples at the final time point (mg/ml), *V*_*n*_ is the volume of the endothelial compartment, *V*_*i*_ is the sample volume, and *A* is the effective area of permeation.

### Measurement of Hair Growth in Mice

Seven-week-old female C57BL/6 mice in the telogen stages of the hair cycle (Müller-Röver et al., 2001) were purchased from Zhejiang Vital River Laboratory Animal Technology Co., Ltd. (Zhejiang, China). After 7-day acclimation in the facility, mice were randomly divided into three groups (*n* = 6). The animal experiment design was adapted from the study of Kwack et al. (2011) but with specific modifications. The dorsal area (3 cm × 4 cm) of each mouse was synchronized to the anagen stage by depilation with a clipper and an electric shaver. At 10 days post-depilation, when all hair follicles in the depilated skin area entered anagen VI, the mice in different groups were wiped with 500 μl of vehicle sample (2% glycerol and 1% PEG 400), AOS alone sample (1% AOS DP2), or AOS formula (1% AOS DP2 with 2% glycerol and 1% PEG 400) daily for 10 days. Then, the mice were sacrificed to obtain skin specimens for morphological and histological observation. For hair length determination, hairs were plucked randomly from the treated dorsal area at 21 day, and the length of 30 hairs per mouse was measured manually. All experimental protocols were conducted in accordance with the guidelines for care and use of laboratory animals as approved by the Institutional Committee on the Care and Use of Animals of Third Institute of Oceanography, NMR (TIO-IACUC-03-2020-06-22).

### Histological Analysis of Skin Specimens

Mouse skin specimens were fixed immediately in 10% neutral formalin, dehydrated using alcohol and xylene, and embedded in paraffin blocks. Five-micrometer sections were stained with hematoxylin and eosin and observed under a light microscope. Hair follicles at distinct hair cycle stages were classified and assigned according to the guideline described by Müller-Röver et al. (2001). To calculate the hair cycle score, hair follicles in anagen V–VI stage, early catagen stage (catagen I–III), mid-catagen stage (catagen IV–V), and late catagen stage (catagen VI–VIII) were arbitrarily attributed a score of 100, 200, 300, and 400, respectively. Fifty hair follicles identified on sections were scored for each mouse, and five mice were used for each group.

### Statistical Analyses

All biological experiments were repeated three times independently. Data are expressed as mean ± standard deviation. GraphPad Prism 5 (GraphPad Software, San Diego, CA, United States) was utilized for analyzing the results. Numerical data were analyzed using one-way analysis of variance. Statistical significance between treatments was analyzed using Student’s *t*-test.

## Results

### Preparation of AOS With Varying DPs

**FIGURE 1:**
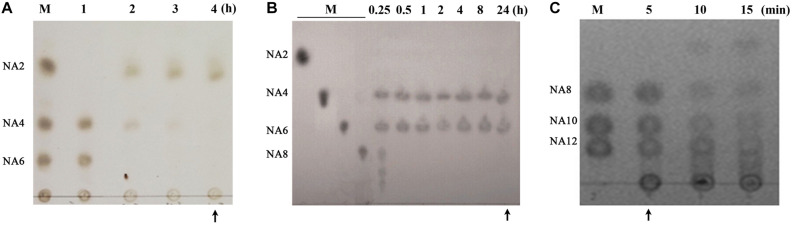
Preparation of algal oligosaccharide (AOS) with varying DP from agar. **(A)** Preparation of AOS (DP2) by enzymatic degradation of AOS (DP4–6) with Aga2660. **(B)** Preparation of AOS (DP4–6) by enzymatic degradation of agar with Aga4383. This picture is adapted from the study of Hou et al. (2015). **(C)** Preparation of AOS (DP8–12) by enzymatic degradation of agar with Aga2133. M, agaroligosaccharide markers; NA2, neoagarobiose; NA4, neoagarotetraose; NA6, neoagarohexaose; NA8, neoagarooctaose; NA10, neoagarodecaose; NA12, neoagarododecaose. The hydrolysate indicated with an arrow in each thin-layer chromatography plate was purified and used in the following experiments.

AOS may exhibit different bioactivities depending on their composition (Gao et al., 2017). Therefore, we first sought to prepare AOS samples with varying compositions from agar. In our previous studies, the agarolytic bacterium *F. pacifica* WPAGA1 was isolated from the deep-sea sediments of the West Pacific Ocean (Xu et al., 2012). A genome sequence analysis of the strain identified three agarases (Aga2133, Aga4383, and Aga2660), which showed distinct endolytic activities toward agar and yielded various hydrolysis products (Hou et al., 2015; Gao et al., 2017). Thus, AOS samples were obtained by agar degradation with the enzymatic action of one or combined agarases (refer to “Materials and Methods” for details). The obtained hydrolysates were then characterized for oligosaccharide composition through TLC analysis (Figure 1). The hydrolysates with different oligosaccharide composition profiles were purified and lyophilized as AOS (DP2) sample (containing NA2, indicated with an arrow in Figure 1A), AOS (DP4–6) sample (containing NA4 and NA6, indicated with an arrow in Figure 1B), and AOS (DP8–12) sample (containing NA8, NA10, and NA12, indicated with an arrow in Figure 1C) for downstream *in vitro* and *in vivo* bioactivity assays.

### Effects of Varying Doses of AOS on the Proliferation of hHFDPCs

**FIGURE 2:**
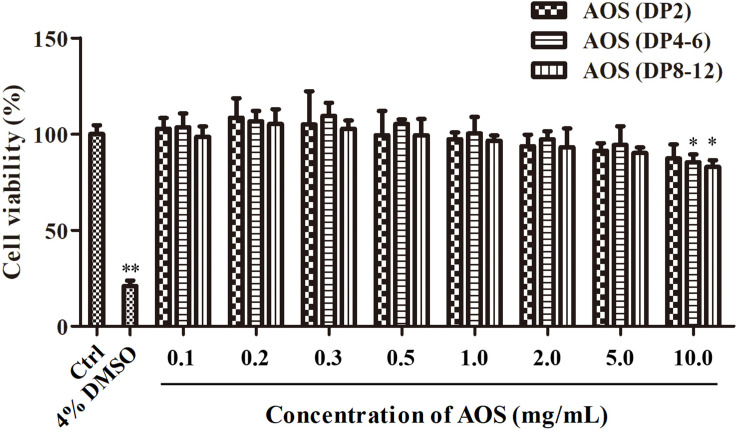
Proliferation of cultured human hair follicle dermal papilla cells (hHFDPCs) treated with algal oligosaccharide (AOS) of varying doses. Different concentrations of AOS were added into cultured hHFDPCs, and cell viability was determined by 3-(4,5-dimethylthiazol-2-yl)-2,5-diphenyltetrazolium bromide assay. As a positive control, 4% dimethyl sulfoxide was included. Columns represent the mean of triplicate assays. Results that are significantly different from those in the blank control group are shown by asterisks (^∗^*p* < 0.05, ^∗∗^*p* < 0.01).

The effects of varying concentrations of AOS on the proliferation of cultured hHFDPCs were investigated to determine the suitable dosage for *in vitro* experiments. Cultured hHFDPCs were treated with various concentrations of AOS with varying DPs (ranging from 0.1 to 10 mg/ml). As shown in Figure 2, treatment of AOS (DP2) up to 10 mg/ml did not influence the proliferation of hHFDPCs, whereas a high dose (10 mg/ml) of AOS (DP4–6) and AOS (DP8–12) inhibited the cell growth rate significantly (*p* < 0.05). Therefore, we selected three gradient doses of AOS (0.2, 1, and 5 mg/ml) for further *in vitro* experiments.

### AOS Antagonized DHT-Induced Cellular Signaling in hHFDPCs

**FIGURE 3:**
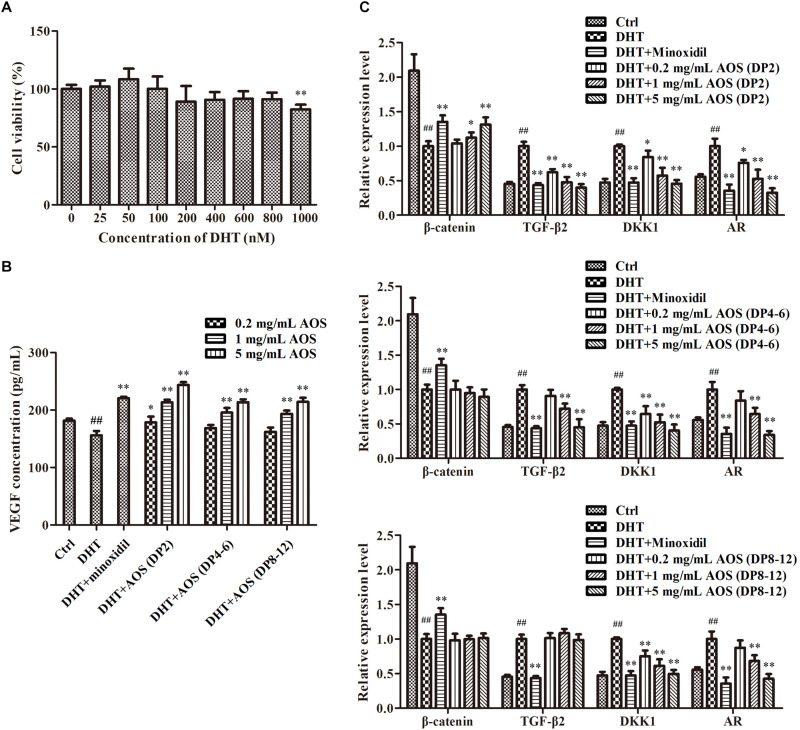
Effects of algal oligosaccharide (AOS) with varying degrees of polymerization (DPs) on vascular endothelial growth factor (VEGF) secretion and hair-growth-related gene expression. **(A)** Influences of dihydrotestosterone (DHT) treatment on the growth of human hair follicle dermal papilla cells (hHFDPCs). Different concentrations of DHT were added into hHFDPCs. After 24 h, cell viability was determined by 3-(4,5-dimethylthiazol-2-yl)-2,5-diphenyltetrazolium bromide assay. **(B)** Effects of AOS with varying DPs on the VEGF secretion of hHFDPCs. Different concentrations of AOS were added into hHFDPCs, and the concentration of extracellular VEGF was determined by enzyme-linked immunosorbent assay. **(C)** Effects of AOS with varying DPs on hair-growth-related gene expression. GAPDH was used as the reference gene for internal control. For all figures, the control group was treated with culture medium. The final concentrations of DHT and minoxidil were 800 nM and 500 μM, respectively. Columns represent the mean of triplicate assays. Significant differences between DHT and control groups are indicated with double hash symbols (^##^*p* < 0.01). Results that are significantly different from those in the DHT group are shown by asterisks (^∗^*p* < 0.05, ^∗∗^*p* < 0.01).

DHT could alter the secretion pattern of dermal papilla by enhancing the release of factors that inhibit keratinocyte growth as well as reducing the secretion of hair-growth-stimulating factors. In this study, DHT treatment with concentrations lower than 800 nM did not show obvious toxicity to hHFDPCs, while 1,000 nM DHT significantly decreased the cell viability (Figure 3A, *p* < 0.01). Thus, 800 nM DHT was used to induce alterations in regulatory paracrine factors in hHFDPCs. As predicted, the addition of 800 nM DHT significantly altered the secretion pattern of regulatory factors in hHFDPCs as evidenced by the significant decrease in the concentration of VEGF (*p* < 0.01) and the significant increase in the expression levels of TGF-β2 (*p* < 0.01), DKK-1 (*p* < 0.01), and AR (*p* < 0.01) relative to the blank control group (Figures 3B,C). Minoxidil was used as a positive control for DHT-induced alteration treatment. As shown in Figures 3B,C, the addition of 500 μM minoxidil effectively ameliorated the DHT-induced alteration of secretion pattern in hHFDPCs. Similarly, the treatment of AOS exhibited apparent mitigation effects against DHT-induced alterations to some extent, as revealed by the partial or complete normalization of the levels of VEGF, TGF-β2, DKK-1, AR, and β-catenin (Figures 3B,C). The mitigation effects of AOS appeared mainly dependent on its dose and DP. In general, the mitigation effects of AOS increased with increasing concentration, but with some exceptions. For example, no obvious difference in the TGF-β2 expression level was found in hHFDPCs treated with different concentrations of AOS (DP 8–12). The minimum concentration required for taking effects differed between AOS with varying DPs. Specifically, either a low or a high concentration of AOS (DP2) could significantly elevate the VEGF concentration (*p* < 0.05), while only a higher concentration of AOS (DP4–6) or AOS (DP8–12) showed similar effects (*p* < 0.05). The results showed that AOS (DP2) exhibited the best mitigation effects because it attenuated all DHT-induced alterations tested, while no mitigation effects were observed for AOS (DP4–6) toward β-catenin and for AOS (DP8–12) toward β-catenin and TGF-β2. In some cases, the mitigation effects of AOS (DP2) were comparable with those of minoxidil. Hence, AOS (DP2) was selected for the following animal experiment.

### AOS Prolonged the Duration of Anagen and Promoted Hair Growth in Mice

**FIGURE 4:**
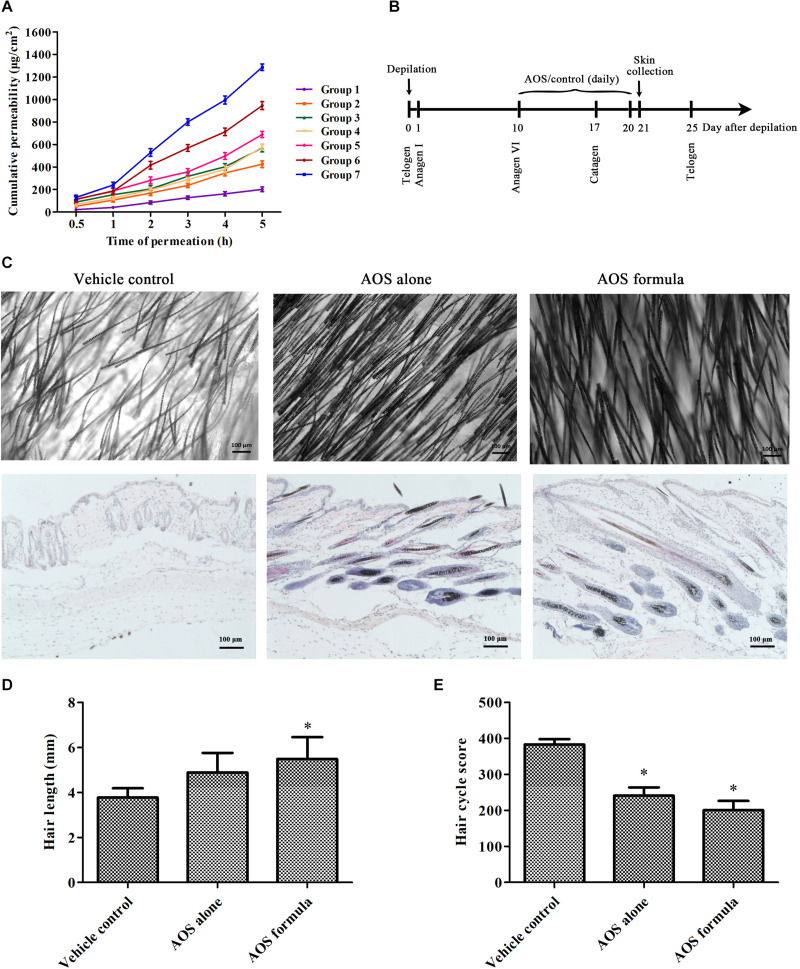
Effects of algal oligosaccharide (AOS) on hair growth of mice. **(A)** *In vitro* transdermal permeation of different AOS penetration-enhancing formula. The abdomen skin of Sprague–Dawley rats was fixed in a Franz diffusion pool. Five hundred microliters of different AOS penetration-enhancing formulas was adopted as the donor solution. The amount of AOS (DP2) in the receiving tank was determined by the dinitrosalicylic acid method. The cumulative permeation per area of the skin (*Q*_*n*_, μg/cm^2^) was calculated. The experiment was repeated three times. **(B)** Scheme of the animal experiment. The back skin of C57BL/6 mice (*n* = 6) was treated with vehicle [2% glycerol and 1% polyethylene glycol (PEG) 400], AOS alone (1% AOS DP2), or AOS formula (1% AOS DP2 with 2% glycerol and 1% PEG 400) at anagen stage for 10 days. **(C)** Effects of AOS on hair follicle growth cycle and hair density. Representative morphological (top) and histological (bottom) images of skin tissue at day 21 are shown. **(D)** Effects of AOS on hair length. The length of randomly plucked hairs (*n* = 30) was measured. **(E)** Calculation of the hair cycle scores. Fifty hair follicles identified on sections were scored for each mouse, and five mice were used for each group. Each stage of the hair cycle has been scored as follows: anagen V–VI = 100, early catagen = 200, mid catagen = 300, late catagen = 400. The value indicates the mean hair cycle score per group (^∗^*p* < 0.05).

Seven groups of AOS permeation-enhancing formula (Table 2) were adopted as the donor solution onto rats’ skin to evaluate the *in vitro* transdermal permeation of AOS (DP2). Figure 4A shows that AOS (DP2) alone barely penetrated into the rat skin within 5 h, although the permeation rate was accelerated at 1 h post-administration. Glycerol or PEG 400 alone enhanced the permeability of AOS by two to three times, while the combination of 2% glycerol and 1% PEG 400 improved the transdermal permeability of AOS upmost, with a value of 6.5-fold at 5 h post-administration. Hence, AOS with 2% glycerol and 1% PEG was applied in the mouse experiment.

Since one of the key features of AGA is shortened anagen period, we further sought to investigate whether AOS exert anagen prolongation effects in mice. The dorsal skin of 7-week-old female C57BL/6 mice was synchronized by depilation. After 10 days, when hair follicles in the depilated skin areas entered the anagen V–VI stage (as evidenced by the skin’s black pigmentation) (Müller-Röver et al., 2001), the depilated areas were treated with vehicle (2% glycerol and 1% PEG 400), AOS alone (1% AOS DP2), or AOS formula (1% AOS DP2 with 2% glycerol and 1% PEG 400) daily for 10 days (Figure 4B). A histological analysis showed more well-formed hair follicles in deep subcutis in both AOS alone and AOS formula group compared with those in the vehicle group, suggesting the extension of the anagen phase and the delayed catagen progression in mice treated with AOS alone or AOS formula (Figure 4C). Determination of the hair cycle score also showed a significantly lower value for both AOS alone and AOS formula group compared with the vehicle group (Figure 4E, *p* < 0.05), demonstrating that AOS significantly delayed the catagen progression of hair follicles. To confirm the hair-growth-promoting effects of AOS, the influences of AOS on hair morphology were further examined. As shown in Figure 4C, an apparent increase in hair density and thickness was observed in both AOS alone and AOS formula group compared with those in the vehicle group (Figure 4C). Besides these, the length of the dorsal hairs treated with AOS formula was significantly increased compared with that of the vehicle group (Figure 4D, *p* < 0.05), while no significant difference of hair length was found between the AOS alone group and the vehicle group (*p* > 0.05), suggesting that the AOS formula exerted better hair-growth-promoting effects than AOS alone.

## Discussion

AOS has received increasing attention because of its physiological and biological functions beneficial for the health of human beings (Han et al., 2016; Gao et al., 2017). However, to our best knowledge, no study has reported on the AGA amelioration activities of AOS. In the present study, we showed that AOS exhibited apparent antagonistic effects in a dose- and DP-dependent manner against DHT-mediated cellular signaling and anagen phase shortening. Since DHT-mediated cellular signaling and the subsequent shortening of the anagen phase play key roles in AGA pathogenesis, AOS may have potential applications in preventing or treating AGA.

Our study showed that AOS significantly ameliorated DHT-induced alteration in dermal papillae cells as revealed by the normalization of several key indices that are important in the pathogenesis of AGA (Figure 3). AOS induced several hair-growth-stimulating factors in dermal papillae cells, such as VEGF and β-catenin. VEGF promotes the generation of capillary vessels in hair follicles, thus nourishing the hair follicles and facilitating hair growth (Yano et al., 2001). VEGF can also induce the proliferation of dermal papilla cells through VEGFR-2-mediated activation of ERK (Li et al., 2012). Some studies have revealed the major role of the Wnt/β-catenin pathway in regulating hair growth (Van Mater et al., 2003), given that β-catenin can interact with AR in an androgen-dependent manner, thereby inhibiting Wnt signaling and hair growth (Chesire and Isaacs, 2002). This finding suggests a functional cross-talk between the AR and the Wnt signaling pathways. On the other hand, AOS suppressed several hair-growth-inhibitory factors, such as AR, TGF-β2, and DKK-1. AR is the binding site of DHT, at which DHT initiates AGA signaling in dermal papilla cells. A previous work reported the increased expression of AR in dermal papillae isolated from balding scalp tissue; thus, localized high levels of AR likely cause the patterned characteristic of balding scalp (Lolli et al., 2017). TGF-β2 suppresses the proliferation of hair follicle epithelial cells by stimulating the synthesis of certain caspases and subsequently activating cell apoptosis. TGF-β antagonists effectively prevent catagen-like morphological changes and promote the elongation of hair follicles *in vitro* and *in vivo* (Hibino and Nishiyama, 2004). DKK-1 was notably up-regulated in the dermal papillae cells of patients with AGA and inhibited hair growth by suppressing the Wnt signaling pathway (Kwack et al., 2012). Collectively, our findings showed that AOS could restore the DHT-induced alteration of regulatory factors in dermal papillae cells and thus may ameliorate AGA to some extent. However, it should be noted that AGA is a complicated disease, and DHT-induced alterations of hair-related genes do not necessarily lead to AGA. In this context, the potential application of AOS in ameliorating AGA may be limited.

The post-natal hair follicle is remodeled during cyclical periods of growth (anagen), regression (catagen), and rest (telogen) (Müller-Röver et al., 2001). The length of the rapid growth stage of anagen is believed to mainly contribute to hair length. One of the key features of AGA is shortened anagen period. Therefore, maintaining the anagen cycle is important for the treatment of AGA. Our results showed that AOS prolonged the anagen phase of hair follicles and promoted the growth of dorsal hair in mice. Similarly, a previous study suggested that minoxidil extended the anagen phase in mice by activating the β-catenin pathway in dermal papillae cells (Kwack et al., 2011).

Although the involvement of androgens in AGA is well established, most of the molecular mechanisms for the pathogenesis of the disorder are unknown (Lolli et al., 2017). In this regard, it is difficult to explore the fundamental mechanisms underlying the regulation effects of AOS on hair-growth-related factors and the anagen cycle. Nevertheless, we speculate that AOS likely exert their effects through their antioxidant activities. Oxidative stress is implicated in AGA pathogenesis. Balding dermal papillae cells from male patients with AGA underwent premature senescence *in vitro* compared with those from occipital scalp when challenged with environmental stress (Bahta et al., 2008; Upton et al., 2015). Bald dermal papillae cells are significantly more sensitive to oxidative stresses, and oxygen significantly alters their morphology, proliferation, migration, senescence, and TGF-β signaling (i.e., secreted higher levels of negative hair growth regulators, namely, TGF-β1 and β2) (Lolli et al., 2017).

Comparisons of the protective effects of AOS with varying DPs revealed a clear pattern, that is, the effects increased with decreasing DP (Figure 3). This finding may be due to the physical phenomenon that compounds with lower molecular weight have better abilities to migrate across the cell membrane. Similarly, AOS with lower DP is supposed to penetrate skin tissue more efficiently, thereby likely to exert better effects than AOS with higher DP when topically applied on skin. Indeed the hair-growth-promoting effect of AOS (DP2) was elevated when it was combined with penetration enhancers that improve its transdermal permeability (Figure 4). Several types of vehicles were reported to enhance the topical delivery efficiency of minoxidil on animals and humans, such as transcutol-containing vesicles (Mura et al., 2011), nanostructured lipid carrier gel (Uprit et al., 2013), and chitosan microparticles (Gelfuso et al., 2011). These types of vehicles may also be useful to improve the transdermal permeability of AOS and should be evaluated for their combined use with AOS in the future.

## Conclusion

In conclusion, AOS significantly ameliorated the DHT-induced alterations of regulatory factors in hHFDPCs and prolonged the anagen phase of hair follicle in mice. Although the fundamental mechanism of the antagonistic effects of AOS against AGA key features is unknown, we speculated that AOS likely exhibited its effects through its antioxidant activities. AOS (DP2) exerted best effects because it likely penetrates the skin tissues and migrates across the cell membranes more efficiently than AOS with higher DP. Therefore, our study demonstrated that marine agarolytic microbes were useful for biodegrading agar to produce AOS with varying DP, which may have potential applications in the prevention and the treatment of DHT-induced AGA.

## Data Availability Statement

The raw data supporting the conclusions of this article will be made available by the authors, without undue reservation.

## Ethics Statement

All experimental protocols were conducted in accordance with guidelines for care and use of laboratory animals approved by the Institutional Committee on the Care and Use of Animals of Third Institute of Oceanography, NMR (TIO-IACUC-03-2020-06-22).

## Author Contributions

RZ and ZC designed the experiments. MJ, Y-LC, XH, and YH performed the experiments, analyzed the data, and wrote the manuscript. All the authors edited and approved the final manuscript.

## Conflict of Interest

XH was employed by the company Aquabrain Biotech (Xiamen) Co., Ltd. The remaining authors declare that the research was conducted in the absence of any commercial or financial relationships that could be construed as a potential conflict of interest.
